# Mechanism of protective actions of sparsentan in the kidney: lessons from studies in models of chronic kidney disease

**DOI:** 10.1042/CS20240249

**Published:** 2024-05-29

**Authors:** Donald E. Kohan, Patricia W. Bedard, Celia Jenkinson, Bruce Hendry, Radko Komers

**Affiliations:** 1 Division of Nephrology, University of Utah Health, Salt Lake City, UT, U.S.A.; 2 Travere Therapeutics, Inc., San Diego, CA, U.S.A.

**Keywords:** Angiotensin II, Endothelin Type A receptor, Endothelin-1, FSGS, IgA nephropathy, Sparsentan

## Abstract

Simultaneous inhibition of angiotensin II AT_1_ and endothelin ET_A_ receptors has emerged as a promising approach for treatment of chronic progressive kidney disease. This therapeutic approach has been advanced by the introduction of sparsentan, the first dual AT_1_ and ET_A_ receptor antagonist. Sparsentan is a single molecule with high affinity for both receptors. It is US Food and Drug Administration approved for immunoglobulin A nephropathy (IgAN) and is currently being developed as a treatment for rare kidney diseases, such as focal segmental glomerulosclerosis. Clinical studies have demonstrated the efficacy and safety of sparsentan in these conditions. In parallel with clinical development, studies have been conducted to elucidate the mechanisms of action of sparsentan and its position in the context of published evidence characterizing the nephroprotective effects of dual ET_A_ and AT_1_ receptor inhibition. This review summarizes this evidence, documenting beneficial anti-inflammatory, antifibrotic, and hemodynamic actions of sparsentan in the kidney and protective actions in glomerular endothelial cells, mesangial cells, the tubulointerstitium, and podocytes, thus providing the rationale for the use of sparsentan as therapy for focal segmental glomerulosclerosis and IgAN and suggesting potential benefits in other renal diseases, such as Alport syndrome.

## Introduction

Evidence collected over three decades supports the use of dual inhibition of the renin–angiotensin–aldosterone (RAAS) and endothelin (ET) systems in the treatment of proteinuric chronic kidney disease (CKD), including rare glomerular disorders. This therapeutic approach has been facilitated by the development of sparsentan, the first dual ET and angiotensin receptor antagonist (DEARA) [1–4].

In parallel with clinical development, studies have been conducted to elucidate the mechanisms of action of this drug and its position in the context of published evidence characterizing the nephroprotective effects of dual ET receptor type A (ET_A_R) and angiotensin receptor type 1 (AT_1_R) inhibition. This review summarizes this evidence. In addition, we discuss the rationale for the use of sparsentan as therapy for focal segmental glomerulosclerosis (FSGS) and immunoglobulin A nephropathy (IgAN) and potential benefits in other renal diseases, such as Alport syndrome (AS).

## Role of ET and RAAS in renal physiology and pathophysiology

Use of sparsentan to treat glomerular diseases requires in-depth understanding of the impact of the ET and RAAS signaling systems in regulating kidney function in health and disease. Basic renal physiology and pathophysiology of these systems have been extensively studied and are the subject of multiple reviews [5–7]. In this section, we briefly summarize the evidence for nephroprotective effects of simultaneous inhibition of these systems and the use of sparsentan.

### RAAS

Angiotensin II (Ang II) and aldosterone, the main effectors of RAAS, have well-known functions in the kidney in health and disease [8,9]. Ang II affects nearly all kidney compartments and cell types through AT_1_R, including hemodynamic actions leading to predominant vasoconstriction of the efferent arteriole and increases in intraglomerular pressure, stimulation of cell growth and extracellular matrix (ECM) production, inflammatory and pro-oxidant activities, and direct effects on podocyte pathophysiology and the pathogenesis of proteinuria [9–14]. Together, these effects ultimately result in glomerulosclerosis and tubulointerstitial fibrosis [8,9]. Additional information on renal Ang II actions is also provided in the context of ET effects below. Traditional physiologic actions of aldosterone are to stimulate sodium reabsorption and potassium excretion in aldosterone-sensitive cells of the distal nephron, thus contributing to the control of blood pressure (BP) and extracellular volume control [15]. Aldosterone also has prosclerotic, fibrogenic, and proteinuric effects [16,17]; thus, the RAAS regulates not only BP but also renal and cardiovascular pathophysiologic processes.

Activation of alternative branches in the RAAS cascade leads to signals that oppose the above-mentioned spectrum of effects of Ang II and aldosterone, which can be viewed as protective in the kidney and cardiovascular system [18]. Among those mechanisms, generation of Ang (1-7) by angiotensin-converting enzyme (ACE) 2 [19] is elevated during AT_1_R or ACE inhibition. Ang (1-7), acting via Mas receptors, has been shown to have vasodilator, antitrophic properties and natriuretic actions [18,20]. Similarly, activation of the type 2 angiotensin II receptor (AT_2_R) by Ang II has protective effects, albeit the physiologic and pathophysiologic relevance of these findings has not been well established. The few known physiologic actions, including natriuresis/diuresis and vasodilation, have been summarized previously by Bader and colleagues [18].

RAS inhibitors (RASis) have been considered standard of care for most causes of CKD [21], including the conditions discussed in this review. To enhance therapeutic efficacy of ACE inhibitors (ACEis) or angiotensin receptor blockers (ARBs), aliskiren was introduced to inhibit the system on the renin level, i.e., at the rate-limiting step of the system. Interestingly, these approaches did not lead to the desired greater treatment effect, particularly in the kidney. Multiple factors might be responsible [22], but the inhibition of the system on the renin level, resulting in the suppression of the aforementioned branches of RAAS considered to be protective in the kidney, may play an important role [18]. However, it should be noted that despite this evidence, RASi represented by ACEis or ARBs remains a mainstay of nonimmunosuppressive nephroprotective treatment [21].

### ET-1 overview

Endothelin-1 (ET-1) is a potent peptide that activates multiple signaling pathways and is strongly implicated in renal pathophysiology. Its synthesis and release are stimulated by numerous factors that trigger or contribute to the development or progression of kidney disease [6,7]. Many actions of ET-1 implicated in renal pathophysiology resemble those of Ang II [23].

Similar to Ang II, ET-1 is involved in the control of renal and glomerular hemodynamics. Actions of ET peptides in the kidney are mediated by ET_A_R and ET_B_ receptors (ET_B_R) [24]; most of the pathophysiological actions of ET-1 in the kidney are mediated via ET_A_R [6]. Much of the evidence for ET-1–induced glomerular injury has been provided by studies demonstrating beneficial effects of ET receptor inhibition or kidney cell–specific ET receptor knockout in models of diseases that lead to glomerulosclerosis [6].

### Effects of ET-1 in the renal vasculature

Functional and binding studies indicate that the renal vasculature expresses both ET_A_R and ET_B_R. In general, ET_A_R are located on renal vascular smooth muscle, while ET_B_R are found on endothelial cells [25,26]. However, vasoconstrictive ET_B_R are found in the renal vasculature of rodent kidneys, while only vasoconstrictive ET_A_R have been reported in rabbit, dog, and human kidneys [27–32]. This species-specific ET receptor expression has led to confusion about the role of ET-1 in the renal vasculature, particularly since most studies on ET-1 vascular actions have been conducted in rats [28,31,32].

ET-1 administered systemically or via the renal artery elicits prompt and sustained whole kidney vasoconstriction [33]. Numerous studies have examined ET-1 actions in the renal microvasculature. Initial studies in rat and rabbit models found that ET-1 constricted isolated afferent and efferent arterioles, with up to 10-fold greater sensitivity in the efferent versus afferent arteriole [34–36]. Subsequent studies in rats using a variety of techniques with intact glomeruli and arterioles (hydronephrotic kidney, isolated perfused kidney, blood-perfused juxtamedullary nephron, micropuncture, and intravital video microscopy of surface glomeruli) largely found that ET-1 resulted in greater constriction in afferent versus efferent arterioles [33,37,38]. However, ET-1–mediated arteriolar constriction in at least two of these systems (hydronephrotic kidney and blood-perfused juxtamedullary nephron) was partly or wholly mediated by ET_B_R [39–42]. In contrast, in the canine kidney, intrarenal ET-1 infusion increased efferent resistance two times more than afferent resistance; of note, this was attributed to direct effects of ET-1 on the microvasculature as well as ET-1 stimulation of other vasoactive mediators (e.g., thromboxane and Ang II) [43].

In healthy humans and animals, ET_A_R do not appear to significantly contribute to BP or renal hemodynamics [43–46], while ET_B_R blockade increases BP and constricts the renal vasculature [44]. However, in healthy individuals given RASi, acute ET_A_R blockade increased renal blood flow and reduced filtration fraction, suggesting that the RAAS and ET systems can interact to regulate renal microvascular function [45,47]. This interaction between RAS and ET_A_R is most apparent in individuals with CKD. Acute ET_A_R blockade increased renal blood flow and decreased filtration fraction in patients with CKD treated with RASi, while acute ET_B_R blockade prevented the renal hemodynamic effects of ET_A_R blockade [44,48]. Similarly, chronic ET_A_R blockade reduced filtration fraction, glomerular filtration rate (GFR), and proteinuria in patients with CKD [49]. Thus, ET_A_R blockade in CKD (in the setting of RAS inhibition) helps restore renal blood flow; this effect is likely mediated by afferent, and relatively greater efferent, arteriolar relaxation.

### Effects of ET-1 in the glomerulus

ET-1 causes mesangial cell contraction and proliferation [50,51] and ECM production [52–55]. This is associated with increased expression and activity of proinflammatory and profibrotic signaling molecules, including nuclear factor κB [55–57], monocyte chemoattractant-1, interleukin-6, adhesion molecules [54,58–60], transforming growth factor ß [52,55,59,61], and connective tissue growth factor [55]. The aforementioned molecules have also been implicated in the renal pathophysiological actions of Ang II [9,12–14] and aldosterone [11,62,63], reflecting extensive overlap and cross talk between the RAAS and ET systems.

ET-1 affects podocytes directly and may play an important role in podocytopathies such as FSGS. ET-1 causes nephrin shedding, loss of synaptopodin, cytoskeletal rearrangement resulting in foot process effacement, and podocyte apoptosis [6,57,59,60,64–66]. These ET-1 actions are mediated via ET_A_R, β-arrestin-1, and Src kinase activation [65]. Additionally, ET-1 activates the Wnt/β-catenin pathway in podocytes [57,65], which is linked to podocyte dysfunction [67]. Treatment with ET receptor antagonists (ERAs) or genetic deletion of ET receptors in models of podocyte injury ameliorated cytoskeletal changes and restored podocyte structural integrity in parallel with reduced proteinuria and glomerulosclerosis [55,65,66,68].

Podocytes also synthesize and secrete ET-1, which has deleterious paracrine effects on adjacent glomerular endothelial cells (GECs); this manifests as mitochondrial dysfunction and production of reactive oxygen species, which in turn impacts the structural integrity of podocytes (as demonstrated in models of FSGS) [66]. In another example of podocyte-GEC cross talk, ET-1 secreted by podocytes acts as a key negative regulator of the GEC glycocalyx, the mesh-like polyanionic carbohydrate structure on the endothelial cell that forms the internal layer of the glomerular filtration barrier. The ET peptide activates heparanase in endothelial cells (and in glomerular macrophages) and causes glomerular endothelial glycocalyx degradation [69–71]. In the context of podocyte and GEC changes, Saleh and colleagues described BP-independent, ET-1–induced, enhanced glomerular permeability to albumin both *in vitro* and *in vivo* [58,72], suggesting that the proteinuric effect is at least partly independent of hemodynamic factors.

### Effects of ET-1 in the tubulointerstitial compartment

The spectrum of proinflammatory and profibrotic ET-1 activity in the tubulointerstitial compartment overlap with the prosclerotic actions in the glomeruli described and can be inhibited by ERAs [55,73]. De Miguel et al. [74] demonstrated that stress induced by ET_A_R stimulation contributes to tubular cell injury and apoptosis. In a recent complex study, Czopek and coworkers [75] showed how multiple ET-1 actions mediated via ET_A_R act in concert to mediate the transition from acute kidney injury to CKD.

In contrast to the anti-natriuretic actions of Ang II or aldosterone, ET-1 acts as a natriuretic peptide [6]. The major renal site of the natriuretic and diuretic actions of ET-1 is the collecting duct, which is also the predominant site of ET-1 tubular production [24,76]. Most evidence indicates that ET_B_R is the main receptor responsible for the natriuretic actions of ET-1 in the nephron; however, observations in mice with double ET_A_R/ET_B_R knockout in the collecting duct, which display more severe hypertension than ET_B_R knockout mice, indicate that ET_A_R contributes to ET-1–induced natriuresis (reviewed by Kohan et al. [33]). Administration of ET_A_R antagonists causes fluid retention in mice, which is prevented by duct-specific knockout of ET_A_R [77]. Importantly, edema and fluid retention can complicate clinical use of selective ET_A_R antagonists [24,78,79], although clinically significant fluid retention has not been reported with sparsentan [4].

### Interactions and cross talk between RAAS and ET-1

In addition to similarities between ET-1 and effectors of RAAS in the pathophysiology of kidney disease, there are also complex interactions and cross talk between these systems. The prosclerotic and inflammatory actions of ET-1 occur as a direct consequence of ET_A_R stimulation and as part of Ang II signaling [9,54,58]. Ang II stimulates ET-1 release and expression in a variety of cell types, including renal cells [80–82]. ET-1 mediates some of the vascular actions of Ang II, both *in vitro* [83,84] and *in vivo* [85], including in the renal circulation [86,87]. In turn, ET-1 stimulates Ang II formation via enhanced action of ACE *in vitro* in pulmonary endothelial cells [88,89]. In addition, ET-1–induced fibronectin synthesis by mesangial cells is reduced by RAAS inhibition [90].

Similar to Ang II, which inhibits renin expression and synthesis in juxtaglomerular cells via AT_1_R, ET inhibits renin expression and release by a Ca^2+^-dependent mechanism [91–93], an effect possibly mediated via both ET_A_R and ET_B_R [94]. However, it remains to be established whether the combination of AT_1_R and ET_A_R inhibition might have additive effect on renin release and expression and whether this process is mitigated by unopposed stimulation of ET_B_R. Although the pathophysiological significance is not yet clear, it should be noted that ET-1 also stimulates aldosterone secretion, as well as zona glomerulosa cell growth and proliferation [95,96].

Interactions of ET-1 with another effector of RAAS, Ang (1-7), have also been described in different experimental settings such as mesenteric arteries in spontaneously hypertensive rats during chronic nitric oxide blockade [97] and in renal vasculature of diabetic spontaneously hypertensive rats [98]. In patients with obesity, Ang (1-7) [1–7] blunted ET-1–induced vasoconstriction in forearm arteries [99]. However, the clinical relevance of these observations remains to be established.

Altogether, there is persuasive evidence for similarities of action and cross talk between RAAS effectors and ET-1 in processes that trigger and perpetuate renal injury. This provides a rationale for potential additive effects of inhibition of both systems in the treatment of kidney disease.

## Evidence supporting combined inhibition of RAAS and ET-1 in the treatment of kidney disease

### Experimental evidence

The concept of dual inhibition of Ang II and ET-1 as nephroprotection has been developing since the 1990s [100,101]. Studies in rodent models have included a passive Heymann nephritis model of idiopathic membranous nephropathy [101] and subtotal nephrectomy [102], a hypertension model involving 5/6 nephrectomy with overexpression of the renin gene [103], and models of diabetic nephropathy [52,61,101]. Across these studies, combining a RASi (using an ARB or ACEi) with an ERA was more effective than monotherapy in attenuating the development and progression of proteinuria, glomerular and tubulointerstitial structural changes, functional deterioration, and molecular changes characteristic of progressive CKD. In uninephrectomized streptozotocin-diabetic rats, the combination restored the number of podocytes, while monotherapies only limited podocyte depletion [52,101].

### Clinical evidence

The antiproteinuric effects of combined RAAS-ERA blockade have been most extensively studied in patients with Type 2 diabetes and nephropathy. Phase 2 and 3 studies demonstrated that the ERAs avosentan and atrasentan lowered proteinuria by 30–50% in patients treated with RASis [79,104–106]. In the RADAR phase 2b trial, atrasentan dose dependently reduced proteinuria in patients receiving stable RASis [79]. In the phase 3 SONAR trial [78], the addition of atrasentan in patients with Type 2 diabetes and nephropathy on the maximal tolerated dose of ARBs or ACEis caused a 35% reduction in the risk of the primary renal outcome (a composite of doubling of serum creatinine, onset of end-stage kidney disease [ESKD], or renal death) over a median follow-up period of 2.2 years.

Until recently, the only available data for the additive effects of ERAs on a background of RASis on proteinuria in patients with nondiabetic CKD (≈50% with IgAN) came from a study by Dhaun et al. [49] (more recent sparsentan data are described in this review). Add-on sitaxsentan (ET_A_R-specific antagonist), but not placebo, lowered proteinuria, BP, and GFR, with unchanged renal plasma flow, resulting in a substantial decrease in filtration fraction in patients treated with RASis. Nifedipine, used for BP control, did not affect proteinuria [49].

## Discovery of dual Ang II and endothelin receptor antagonists

The experimental and clinical studies reviewed thus far used a combination of two individual inhibitors of the RAAS and ET systems. As one molecule with high affinity for both AT_1_ and ET_A_ receptors, sparsentan represents a new approach [2].

**Figure 1:**
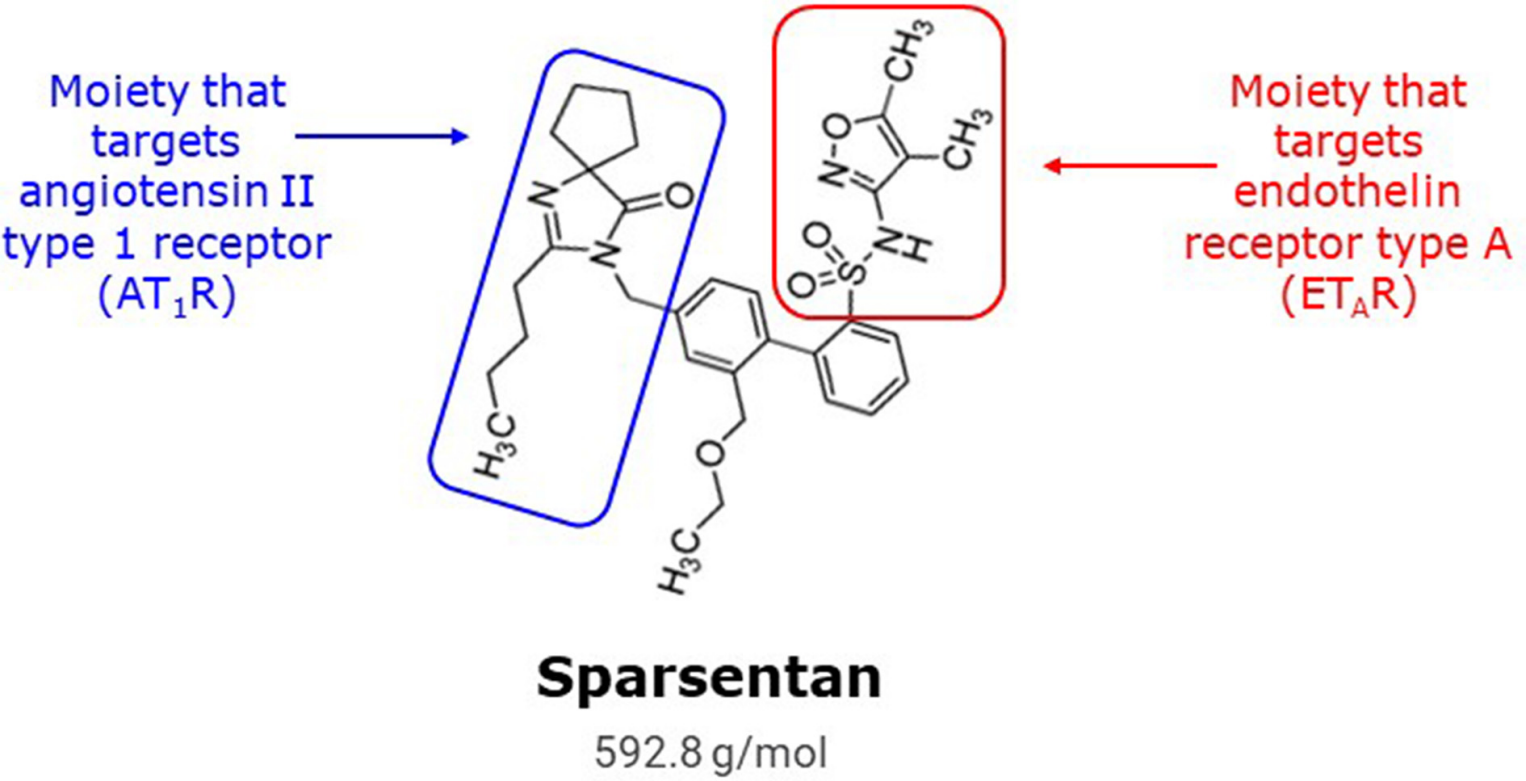
Chemical structure of sparsentan Chemical structure of the dual endothelin angiotensin receptor antagonist (DEARA), sparsentan, showing the key structural elements of the molecule responsible for antagonism of each receptor, ET_A_R and AT_1_R (adapted from [2]). Adapted from Murugesan N, et al. (2005) *J. Med. Chem*. **48**(1):171–179. Copyright © 2005, American Chemical Society.

The discovery of dual angiotensin and endothelin receptor antagonists (i.e., single molecules that can inhibit both receptors) was based on recognition of a biphenyl core that some ERAs had in common with several ARBs, including irbesartan [107]. It was hypothesized that merging the active moieties of these two classes of antagonist via this shared biphenyl core would yield a compound with activity for both receptors. This strategy led to the design and synthesis of a novel class of orally administered antagonists of both AT_1_R and ET_A_R in a single compound (Figure 1 and Table 1).

**Table 1:** *In vitro* binding affinities and *in vivo* pressor inhibition of orally administered ET_A_R and AT_1_R antagonists through development and optimization

Target receptor (s)	Antagonist name	ET_A_R *K*_i_ (nM)	AT_1_R *K*_i_ (nM)	ET_B_R *K*_i_ (nM)	AT_2_R *K*_i_ (nM)	Oral big ET-1 pressor (30 µM/kg) (AOC units)*	Oral Ang II pressor (30 µM/kg) (AOC units)*
ET_A_R	BMS-193884	1.4^†^	>10,000^†^	-	-	10,100^*^	Inactive^*^
AT_1_R	Irbesartan	>10,000^†^	1.1^†^	-	-	Inactive^*^	11,940^*^
ET_A_R & AT_1_R	Unnamed biphenylsulfonamide	39^†^	ND^†^	-	-	-	-
ET_A_R & AT_1_R	BMS-248360	1.9^†^	10^†^	-	-	7,900^*^	6,000^*^
ET_A_R & AT_1_R	BMS-346567 (sparsentan)	9.3^*^	0.8^*^	>10,000^*^	>10,000^*^	15,800^*^	18,600^*^

The chemical structures of the mono-selective antagonists BMS-193884 and irbesartan were merged to produce the first-generation dual ET_A_R/AT_1_R antagonist, an unnamed biphenylsulfonamide. Optimization regained the original target ET_A_R activity (BMS-248360). Further optimization retained dual specificity while improving bioavailability in dog and monkey and oral pressor inhibition in rat (BMS-346567/sparsentan, bioavailability data not shown).

Abbreviations: ‘-’ = not shown; AOC, area over curve, indicating potency and duration of action at equimolar doses; AT_1_R, AT_1_ receptor; ET_A_R, ET receptor type A; *K*_i_, inhibitory constant; ND, not determined.

* Murugesan 2005 [107].

† Murugesan 2002 [2].

Subsequent rational drug design to improve pharmacokinetics while maintaining dual receptor potency resulted in the discovery of sparsentan (compound #7) [2]. Receptor affinity constants (*K*_i_) were determined using radioligand binding assays. The affinity of sparsentan was 0.8 nM for human AT_1_R and 9.3 nM for human ET_A_R, whereas the affinity toward AT_2_R and ET_B_R was negligible (>10 µM) [2]. For comparison, the *K*_i_ of irbesartan for AT_1_R was 1.1 nM [1]. The dual action of sparsentan was demonstrated *in vivo* by its ability to attenuate the pressor effects of intravenous infusions of either Ang II or big ET-1 in rats [107]. Moreover, sparsentan was more effective in reducing BP than an equimolar dose of irbesartan in models of hypertension such as spontaneously hypertensive rats [1]. Leach and colleagues [108] reported similar sparsentan affinity constants for AT_1_R (*K*_i_ = 0.9 nM) and ET_A_R (*K*_i_ = 13 nM) and also showed selectivity over ET_B_R (*K*_i_ = 6582 nM).

## Sparsentan in IgAN

### Overview of IgAN

IgAN is the most common diagnosis made using kidney biopsies performed to evaluate glomerular disease in children and adults [109]. For clinical presentation and current treatment options, readers are referred to recent reviews in the field [110]. IgAN is now viewed as a multiple-hit disease process. The initial step is the formation of hypo-galactosylated IgA1 molecules (Gd-IgA1). This leads to the synthesis of anti–Gd-IgA/IgG antibodies and the formation of immune complexes, the second and third hits. The immune complexes are deposited in the mesangial region of the kidney, triggering inflammation and progressive glomerular injury [111–113]. Generation of Gd-IgA1 occurs in the respiratory and gastrointestinal tracts [114–117]. Recent evidence has linked IgAN to the activity of the complement system [118].

RASis were the standard of care for patients with IgAN and proteinuria; however, even with the addition of sodium-glucose cotransporter-2 inhibition or steroids (systemic or gut targeted), significant residual proteinuria, renal functional deterioration, and continued risk of kidney failure occurs [110,119,120]. Activation of the renal ET system in IgAN is supported by several lines of evidence. In addition to a previously mentioned study by Dhaun et al. [49] indicating renal ET system activation in IgAN, Lehrke et al. [121] reported that renal ET-1 gene expression was significantly higher in patients with higher-grade proteinuria compared with patients with lower-grade proteinuria or control participants. Elevated renal ET gene expression was also reported by Tycova et al. [122] in patients with IgAN and was associated with an increased risk of disease progression.

### Effect of sparsentan in animal models of IgAN

Sparsentan has been tested in an IgAN mouse model in which engineered immune complexes formed from human Gd-IgA1 and recombinant IgG autoantibody were injected into nude mice to induce glomerular injury mimicking human IgAN. Treatment with sparsentan attenuated both mesangial hypercellularity and proliferation (Ki67 immunoreactivity) in engineered immune complex–treated mice compared with those that received vehicle [123]. RNA sequencing analysis of kidney biopsies taken at the end of the study revealed that up-regulation of key inflammatory and proliferative genes and pathways was markedly attenuated by sparsentan treatment, including complement genes (*C1q* [complement C1q]), integrin components (*Itgb2* [integrin subunit beta 2]), members of the MAP kinase family (*MAPK1/3/8/14*), and Fc receptor elements (*Fcer1g* [Fc epsilon receptor Ig]) (date on file; Travere). Moreover, translatability of the transcriptional findings for the immune and inflammatory pathways was supported by partial overlap between differentially expressed murine and human genes in patients with IgAN [124].

The IgAN-prone gddY mouse model spontaneously develops albuminuria by 8 weeks of age; by 12 weeks of age, expression of messenger RNA (mRNA) for ET-1, ET_A_R and AT_1_R, and inflammatory pathway genes is up-regulated compared with healthy Balb/c control mice [125]. Sparsentan was compared with an ARB (losartan) in this model. Compared with control gddY mice, mice that received sparsentan and losartan had equivalently reduced BP and ameliorated increases in mRNA for ET-1, ET_A_R and AT_1_R, and inflammatory genes. However, sparsentan reduced albuminuria more rapidly than losartan. Moreover, sparsentan protected the kidney from glomerulosclerosis and glycocalyx and podocyte loss to a far greater extent than losartan [125].

Sparsentan has also exerted beneficial effects in the rat anti-Thy1 model [126]. Although not an IgAN model per se, the model exhibits mesangial proliferation and glomerular pathologies that overlap with IgAN, resulting from the binding of anti-Thy1 to the Thy1 antigen on the surface of mesangial cells and subsequent complement activation and mesangiolysis, leading to up-regulation of ET-1 mRNA expression. Sparsentan dose dependently attenuated the increase in proteinuria and mesangial cell proliferation (Ki67 immunoreactivity) and activation (α-smooth muscle actin protein expression) and provided protection from macrophage infiltration, glomerulonephritis, and interstitial myofibroblast activation (α-smooth muscle actin protein expression) (Table 2).

**Table 2:** Summary of disease mechanisms impacted by sparsentan in nonclinical models

	Nonclinical model					
Disease mechanism	gddY IgAN [125]	EIC model of IgAN [123,124]	Mesangioproliferative GN (anti-Thy1) [126]	Adriamycin rat FSGS [144]	TRPC6-Tg FSGS [138,139]	Alport mouse [154]
**Proteinuria**	√	Not tested	√	√	√	√
**Glomerulosclerosis**	√	NA	√	√	√	√
**Podocytes**	√	Not tested	Not tested	√	√	√
**Mesangial cells**	NA	√	√	NA	√	NA
**mGFR**	Not tested	Not tested	Not tested	Not tested	√	√
**Glycocalyx**	√	Not tested	Not tested	√	√	Not tested
**Glomerular basement membrane**	Not tested	Not tested	Not tested	√	Not tested	√
**Vasoconstriction**	Not tested	Not tested	Not tested	Not tested	√	Not tested
**Inflammation**	√	√	√	√	√	√
**Tubules/interstitium**	Not tested	Not tested	√	Not tested	√	√
**Extracellular matrix**	Not tested	Not tested	√	Not tested	Not tested	√

Abbreviations: EIC, engineered immune complex; FSGS, focal segmental glomerulosclerosis; GN, glomerulonephritis; IgAN, immunoglobulin A nephropathy; mGFR, measured glomerular filtration rate; NA, not applicable; Tg, transgenic.

## Sparsentan in FSGS

### Overview of FSGS

FSGS is a renal histological lesion triggered by podocyte injury and characterized by segmental accumulation of glomerular ECM, resulting in glomerular scarring and capillary obliteration [127,128]. A range of heterogeneous clinical conditions and stimuli lead to podocyte injury and consequently to FSGS-type lesions [129], which are classified as primary, genetic, secondary, or forms with unknown cause [110]. Primary FSGS has no identifiable cause but may result from the actions of putative circulating permeability factors that cause podocyte injury [129]. The rapidly expanding list of genetic causes includes mutations in genes encoding proteins required for normal podocyte structure or function. Heterozygous mutations of *Col4a3*, *Col4a4*, and *Col4a5* genes or high-risk *APOL1* alleles are associated with increased risk of FSGS and lead to the characteristic histopathological lesions [128–131]. There are numerous secondary forms of FSGS. Renal lesions in secondary FSGS are due to adaptive structural-functional responses (i.e., a mismatch between metabolic and hemodynamic load and glomerular capacity), such as in patients with congenital or acquired reduction of renal mass, obesity, metabolic derangements, other antecedent diseases, drug use, or infections [128,129].

The presentation of FSGS features varying levels of proteinuria, including severe nephrotic syndrome [132–134]. RASis are used in most patients with FSGS for their antiproteinuric and nephroprotective actions [135,136]. Glucocorticoids remain the mainstay of treatment for patients with FSGS presenting with nephrotic syndrome or rapid loss of kidney function [110]. Additional immunosuppressive agents, such as calcineurin inhibitors, mycophenolate mofetil, or rituximab, are used in patients with steroid-resistant disease to achieve remission and clinically meaningful reduction in proteinuria, albeit with limited success.

Considering the multiple roles of Ang II and ET-1 in podocyte pathophysiology and the development of glomerulosclerosis, as previously discussed, dual ET_A_R and AT_1_R inhibition seems to be a logical approach for treatment of most forms of FSGS. Moreover, a recent study demonstrated an increased percentage of glomeruli with ET_A_R-positive endothelial cells in patients with primary FSGS compared with healthy control participants. Glomerular ET_A_R-positive endothelium was strongly associated with nephrin loss, glomerular markers of oxidative stress, and proteinuria, further supporting the role of the ET system in the development of FSGS [137].

### Effect of sparsentan in models of FSGS

Transgenic mice overexpressing wild-type TRPC6, specifically in podocytes, develop kidney disease similar to human FSGS. In untreated FSGS transgenic mice, the detectable signs of ongoing FSGS pathology include segmental elevations in podocyte calcium, the development of parietal podocytes and adhesions between parietal and visceral layers of Bowman’s capsule, and albumin leakage through the glomerular filtration barrier. In studies by Gyarmati et al. [138,139], treatment with sparsentan for 6 weeks ameliorated glomerulosclerosis and tubulointerstitial fibrosis in this model to a similar degree as losartan. In contrast, sparsentan was more effective in preservation of podocyte number and inhibition of podocyte calcium influx. Multiphoton imaging in intact living kidneys demonstrated greater afferent and efferent arteriole diameters, increased single-nephron GFR and blood flow, and reduced urine albumin/creatinine ratio and albumin sieving coefficient in mice that received 6 weeks of sparsentan treatment compared with losartan-treated TRPC6 mice [138,139]. Parallel experiments demonstrated that the beneficial effects of sparsentan in glomeruli in this model were linked to attenuation of mitochondrial stress in podocytes, restoration of the integrity of the glomerular endothelial surface layer (glycocalyx), and reduction in CD44^+^ immune cell homing [139].

Further studies by this group focused on tissue remodeling. In this model, sparsentan increased the frequency of larger multi-cell clones in both the renin and endothelial lineage in glomeruli and arterioles. Losartan had a similar effect, but with a reduced magnitude compared with sparsentan. In addition, various renal cortical and medullary tubule segments including cells of the proximal tubule, the distal convoluted tubule and the collecting duct also showed active cellular (clonal) remodeling in response to sparsentan, and, to a lesser extent, losartan [139]. The renin cell lineage is known to have the ability to function as progenitor cells for parietal and epithelial cells as well as podocytes during glomerular structural and functional repair [140,141]. Altogether, this multidirectional evidence supports sparsentan's potential to preserve glomerular structural integrity in addition to its hemodynamic actions.

Of note, several studies have demonstrated concentric hypertrophy of intrarenal arterioles caused by multiclonal expansion of renin cells both in mice and humans in response to RAS inhibition, resulting in glomerular blood flow obstruction, downstream ischemia, and even development of renal fibrosis [142,143]. Our observations in TRPC6 mice treated with sparsentan indicate opposite effects on glomerular hemodynamics and structural benefit [138,139]. Whether these opposite effects are mediated via concomitant ET receptor inhibition remains to be established.

The adriamycin (ADR) murine model is characterized by rapid podocyte injury and proteinuria followed by glomerulosclerosis, tubulointerstitial inflammation, and fibrosis with lesions reflective of human FSGS. Sparsentan treatment attenuated proteinuria and podocyte loss, maintained glomerular basement membrane width, protected the glycocalyx, and reduced glomerular macrophage infiltration [144]. Data from an ADR rat study were used to establish a disease and treatment profile using differential gene expression mapped to data from a cohort of patients with FSGS in the NEPTUNE registry [145]. A series of genes were identified that showed dysregulated expression in the diseased rats and expression changes that were reversed by sparsentan treatment. Human orthologs of these genes showed altered expression in the FSGS cohort compared with healthy controls that correlated with disease severity as measured by proteinuria and estimated glomerular filtration rate (eGFR).

An interesting novel mechanism of action for sparsentan was recently published [146]. The authors found increased numbers of CD8^+^ tissue-resident memory T (T_RM_) cells in the glomeruli and tubulointerstitial compartment in the ADR model and in patients with clinical FSGS, as well as in models of and patients with lupus nephritis and Type 2 diabetes. Levels of interleukin-15 (IL-15) were increased in ADR mice and correlated with albuminuria. IL-15 promoted renal CD8^+^ TRM development and interferon‐γ (IFN-γ) secretion. Inhibition of IL-15 with an antibody reduced renal CD8^+^ TRM formation and IFN-γ generation, with a beneficial impact on albuminuria, glomerulosclerosis, podocyte morphology, and nephrin/podocin mRNA expression. Considering their parallel observation that IL-15 in renal cells can be induced by Ang II/ET-1, the authors treated ADR mice with sparsentan. The intervention mimicked the IL-15 inhibition described, suggesting a new potential anti-inflammatory and podocyte-protective mechanism for the drug [146].

## Sparsentan in Alport syndrome

### Overview of AS

AS is an inherited progressive form of glomerular disease that is often associated with extra-renal manifestations, including sensorineural hearing loss and ocular abnormalities [147–149]. AS is a primary basement membrane disorder that results from mutations in genes encoding the alpha-3-5 chains of type IV collagen. In early childhood, hematuria and microalbuminuria are detected. Over time, proteinuria, nephrotic syndrome, hypertension, and progressive renal insufficiency develop at a rate that depends on the type of mutation, but ESKD may occur as early as the third decade of life in patients with X-linked or autosomal recessive disease [147–149].

The treatment of AS is currently based on RASi initiation at the time of diagnosis, especially in patients with proteinuria [150–152]. Data suggest that ACEis postpone ESKD and improve life expectancy in patients with AS, although disease progression continues [151]. A role for ET-1 has also emerged in AS. Dufek and colleagues showed that activation of CDC42/RAC1 leads to mesangial filopodial invasion of glomerular capillaries, resulting in the deposition of mesangial proteins, including laminin α2, in the glomerular basement membrane in a mouse model of autosomal recessive AS (COL4A3 knockout on an SV/129 background) [153]. Laminin α2 directly injures podocytes by activating focal adhesion kinase and nuclear factor κB. Importantly, ET_A_R blockade prevented mesangial filopodial invasion and ameliorated glomerular disease in these AS mice.

### Effect of sparsentan in experimental AS

Sparsentan and losartan were compared in the COL4A3 knockout mice studied by Dufek et al. [153], in which proteinuria and renal pathology typically begin at 5 weeks of age and lifespan is approximately 10 weeks [154] (Table 2). The mice also develop susceptibility to hearing loss when exposed to metabolically stressful noise between 8 and 9 weeks of age. Sparsentan administered from 4 to 7 weeks of age significantly delayed the onset of glomerulosclerosis and interstitial fibrosis, proteinuria, and GFR decline (assessed using a transdermal device to follow the change in fluorescence of fluorescein isothiocyanate–labeled sinistrin over time). Sparsentan attenuated glomerular basement membrane dysmorphology and foot process effacement, blunted mesangial filopodial invasion into the glomerular capillaries, and increased lifespan to a greater extent than losartan despite equivalent BP changes. Sparsentan also lessened changes in the mRNA expression of proinflammatory (*Ccr2* and *IL-1β*), profibrotic (*Mmp2* and *Serbp1*), proliferative (*Myc*), and adhesion pathway genes (Itga2) that were associated with AS. Notably, sparsentan, but not losartan, prevented accumulation of ECM in the strial (cochlear) capillary basement membranes in the inner ear and reduced susceptibility to hearing loss. Improvements in lifespan and in renal and strial pathology were also observed even when sparsentan was administered following development of renal pathology in mice at 5 weeks of age.

## Clinical experience with sparsentan

Sparsentan was approved in 2023 by the US Food and Drug Administration to reduce proteinuria in adults with primary IgAN who are at risk of rapid disease progression [155,156]. This approval was based on the PROTECT study, a phase 3 clinical trial in which 404 adult patients with IgAN with persistent proteinuria of >1 g/day at high risk for kidney failure despite maximal tolerated ACE or ARB therapy were randomized to either sparsentan or irbesartan. The interim analysis demonstrated that patients treated with sparsentan experienced rapid and sustained proteinuria reduction compared with those who received a maximum tolerated dose of irbesartan (−49.8% vs −15.1%, respectively; *P*<0.0001). Sparsentan was well tolerated, with a safety profile comparable to that of irbesartan [157].

The 2-year data from the PROTECT trial demonstrated marked and sustained reductions in proteinuria, associated with slower kidney function decline, in sparsentan-treated compared with irbesartan-treated patients with IgAN [4].

Sparsentan has also been tested in ongoing phase 2 and 3 randomized controlled trials in patients with FSGS. Both the phase 2 DUET and phase 3 DUPLEX trials demonstrated a significantly greater and sustained reduction in proteinuria in patients with FSGS treated with sparsentan compared with those treated with irbesartan [158,159], although in DUPLEX the effect on eGFR slopes over 2 years between the sparsentan and irbesartan arms was not statistically significant [3,156].

In general, treatment with sparsentan has been well tolerated in both FSGS and IgAN studies [4]. In previous studies investigating ERA, the main safety signal of concern has been fluid retention causing edema or even heart failure (RADAR [79], SONAR [78], and ASCEND [160]). Based on the post hoc analysis of the RADAR and RADAR/JAPAN studies with atrasentan, lower eGFR, higher BP, and higher inhibitor dose have been identified as risk factors for fluid retention in ERA-treated patients [161].

A striking feature of the clinical studies with sparsentan is the lack of clinically significant edema even at doses escalated to 800 mg daily. The PROTECT study shows that sparsentan has actions qualitatively and quantitatively different from a simple increase in angiotensin pathway suppression, supporting ET_A_R engagement in humans. These actions include strong proteinuria reduction with minimal effects on BP, no exacerbation of hyperkalemia, changes in diastolic BP greater than systolic BP, and a lack of additional acute reductions in eGFR [4,157]. A detailed analysis of the 24-h pharmacokinetic profile and estimation of the ET_A_R and AT_1_R occupancies of sparsentan at steady-state 400-mg dosing is shown in Figure 3. ET_A_R occupancy is in the range of 60–90% at all times, while AT_1_R occupancy is always above 95%. Substantial ET_A_R antagonism is therefore achieved, always accompanied by even greater AT_1_R blockade. This obligatory relationship (as sparsentan possesses dual antagonism in a single molecule) cannot be guaranteed with a single-target ERA combined with an ARB, especially as the dose is increased, and may underlie the edema safety profile of sparsentan. Other strategies have been employed to reduce the risk of edema with ERA administration (dose reduction [162], patient stratification [78], and combination therapy with sodium-glucose cotransporter-2 inhibitors [163,164]) with varying degrees of success. Additional data are needed to elucidate which strategies are best suited to which patient populations.

## Summary of mechanisms of nephroprotective actions of sparsentan

- The protective effects of sparsentan in models of kidney disease corroborate abundant evidence collected over the past decades on the renal pathophysiology of renin–angiotensin and ET systems.
- In mesangial cells, sparsentan exerts antiproliferative effects and inhibits production of proinflammatory and profibrotic cytokines and ECM proteins, resulting in amelioration of corresponding glomerular lesions such as mesangio-proliferative glomerulonephritis or glomerulosclerosis [123,126].
- In glomerular endothelial cells, sparsentan preserved glycocalyx integrity in several models, a mechanism likely to contribute to its antiproteinuric effect [125,138,139,144].
- In podocytes, sparsentan inhibits Ca^2+^ flux, an established marker of podocyte injury, leading to preservation of nephrin and podocin expression, reduced oxidative stress, attenuation of foot process effacement, and maintenance of the glomerular basement membrane. Given the ability of sparsentan to inhibit IFN-γ [146], which has been shown to be a key regulator of *APOL1* synthesis, the drug may be particularly useful in individuals with FSGS and *APOL1* risk alleles.
- In the tubulointerstitial compartment, sparsentan ameliorated tubulointerstitial fibrosis in association with normalization of proinflammatory cytokine mRNA and protein levels, profibrotic mediators, ECM proteins, and complement components [124,125,144].
- In the renal vasculature, sparsentan likely exerts protective glomerular hemodynamic effects through mitigation of Ang II– and ET-1–mediated efferent arteriolar constriction [138,139].

**Figure 2:**
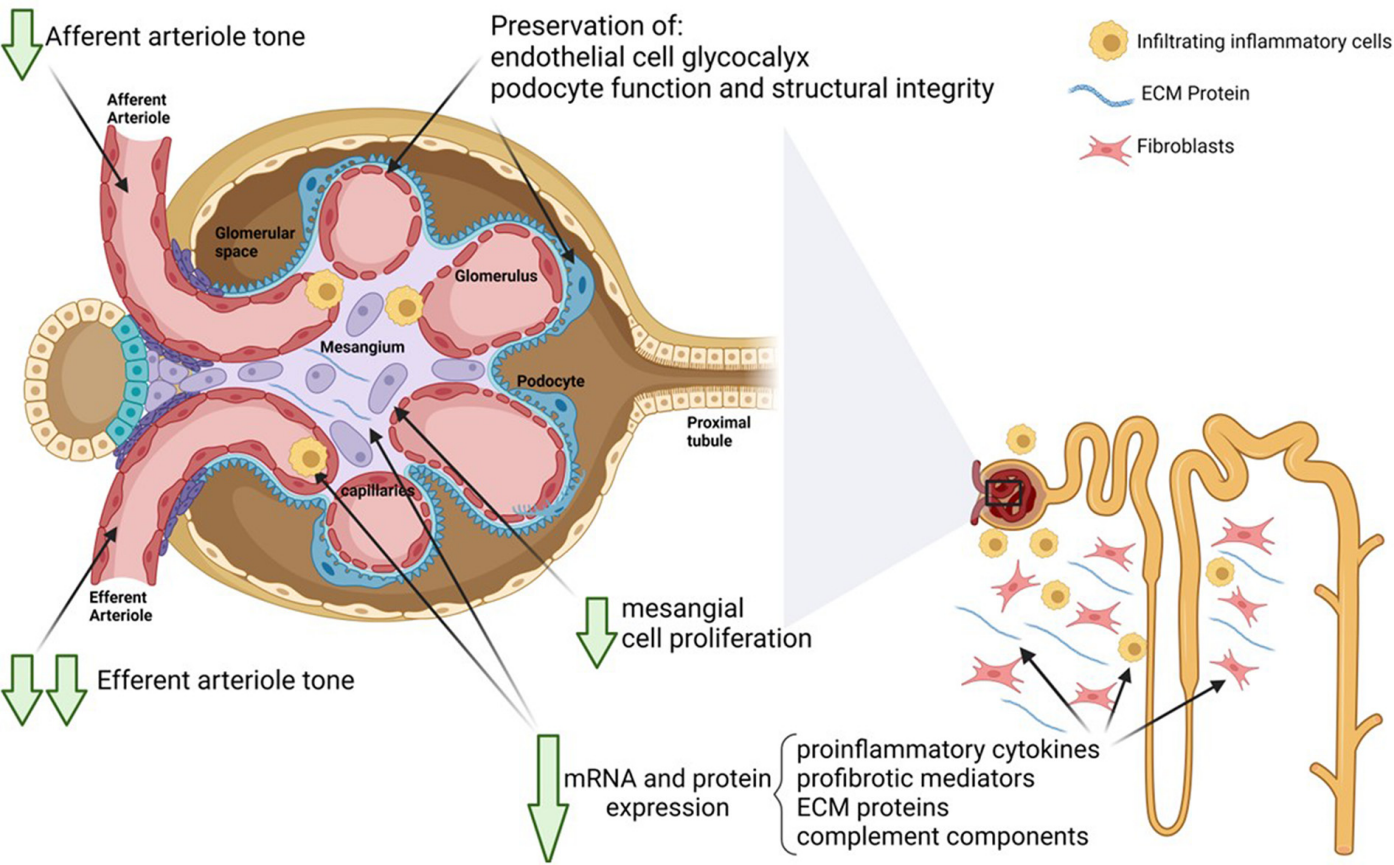
Mechanisms of action of sparsentan in the kidney Schematic presentation of main actions of sparsentan in the kidney associated with long-term nephroprotective effects as observed in preclinical studies. These actions include anti-inflammatory and antifibrotic effects both in glomeruli and the tubulointerstitial compartment, podocyte protection, and preservation of the glomerular glycocalyx, in parallel with beneficial effects on glomerular hemodynamics. Created with Biorender.com by Wilmelenne Clapper. ECM, extracellular matrix; mRNA, messenger RNA.

Taken together, the growing body of preclinical data suggest numerous direct cellular, and resulting structural, actions of sparsentan contributing to nephroprotection (Figure 2). The SPARTAN study (NCT04663204), a single-arm interventional study of sparsentan in patients with incident IgAN, is collecting kidney biopsies at baseline and after 24 weeks of treatment and may further inform whether these data translate to the clinical setting.

**Figure 3:**
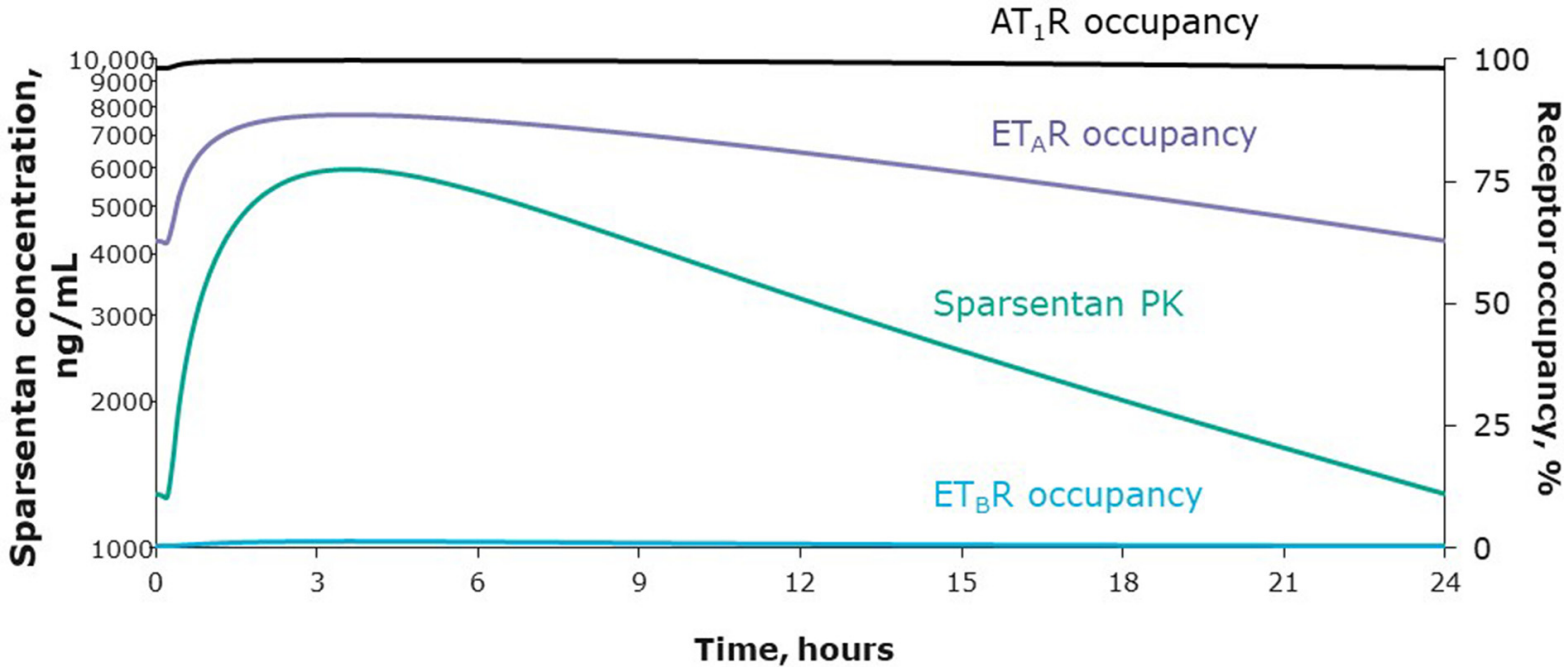
Sparsentan ET_A_R and ET_B_R occupancies (right axis) and plasma concentration* (left axis) over 24 h for a single, daily, 400-mg oral dose at steady-state in the PROTECT study (adapted from [165]) Steady-state PK parameters calculated using population PK values for sparsentan 400 mg in the PROTECT study were used to estimate diurnal changes in receptor occupancy. Sparsentan AT_1_R occupancy (>95%) consistently exceeds ET_A_R occupancy (>60% and <90%) over a full 24-h period. ET_B_R occupancy never exceeds 2%. *PK data are based on population PK model prediction for a patient with IgAN. AT_1_R, angiotensin II receptor type 1; ET_A_R, endothelin receptor type A; ET_B_R, endothelin receptor type B; PK, pharmacokinetics.

## Conclusion

ET and Ang II are important mediators of renal disease. The two systems closely interact to exert additive pathophysiological effects through cross talk involving multiple signaling pathways that impact the renal vasculature, glomerulus, and tubulointerstitium. Dual blockade of AT_1_ receptors and ET_A_R exerts anti-inflammatory, antiproliferative, antifibrotic, and cell protective actions in many forms of kidney disease. Sparsentan, a dual ET and angiotensin receptor antagonist, has been shown in cells, in animal models, and clinically to reduce pathophysiological processes and ameliorate renal injury. As such, sparsentan holds significant promise as a renoprotective agent in CKD.

## Data Availability

N/A - All supporting data are included within the main article.

## Abbreviations

ACE: angiotensin-converting enzyme
ACEi: ACE inhibitor
ADR: adriamycin
Ang II: angiotensin II
ARB: angiotensin receptor blocker
AS: Alport syndrome
AT_1_R: angiotensin receptor type 1
BP: blood pressure
CKD: chronic kidney disease
DEARA: dual endothelin angiotensin receptor antagonist
ECM: extracellular matrix
Gd-IgA1: hypo-galactosylated IgA1 molecules
GEC: glomerular endothelial cells
eGFR: estimated glomerular filtration rate
ERA: endothelin receptor antagonist
ET_A_R: endothelin A receptor
ET_B_R: endothelin B receptor
ET-1: endothelin-1
FSGS: focal segmental glomerulosclerosis
GED: glomerular endothelial cells
IFN-γ: interferon‐γ
IgAN: immunoglobulin A nephropathy
Ki: affinity constants
RAAS: renin–angiotensin–aldosterone system
RASi: RAS inhibitor
